# A Case of Mandibular Osteomyelitis Occurring in a Patient With Parry-Romberg Syndrome

**DOI:** 10.7759/cureus.99021

**Published:** 2025-12-12

**Authors:** Riyu Koguchi-Yoshida, Michita Tayama, Hiroshi Kaneko, Haruhisa Watanabe, Tadahiro Iimura, Yutaka Maruoka

**Affiliations:** 1 Department of Pharmacology/Department of Oral Diagnosis and Medicine, Hokkaido University, Faculty and Graduate School of Dental Medicine, Sapporo, JPN; 2 Department of Oral Surgery, National Center for Global Health and Medicine, Tokyo, JPN; 3 Division of Rheumatic Disease, National Center for Global Health and Medicine, Tokyo, JPN; 4 Department of Pharmacology, Hokkaido University, Faculty and Graduate School of Dental Medicine, Sapporo, JPN

**Keywords:** facial atrophy, jaw osteomyelitis, osteonecrosis of the jaw, parry romberg syndrome (prs), scleroderma

## Abstract

Progressive hemifacial atrophy, also known as Parry-Romberg syndrome (PRS), is an idiopathic disorder characterized by progressive unilateral facial depression caused by atrophy of the skin and subcutaneous tissue. We present a case of mandibular osteomyelitis occurring in a patient with this condition, together with a review of the literature. The clinical findings in this case suggest that autoimmune disease and trauma are involved in contributing factors to disease progression. Possible involvement of neurovascular and neuroskeletal dysregulations in the pathogenesis of PRS is also discussed.

## Introduction

Parry-Romberg syndrome (PRS) is a rare disorder characterized by unilateral hemifacial atrophy that gradually advances. It was first reported by C. Parry and M. Romberg [1,2]. In progressive cases, atrophy affects not only the skin but also the underlying tissues, such as fat, muscle, bone, and cartilage, resulting in severe facial asymmetry [3]. More severe cases indicate that atrophy can progress to the other half of the face or to the neck [4]. Most cases are sporadic, presenting at birth, usually manifesting in childhood or adolescence. The age of onset ranges from one to 50 years, with approximately 75% occurring before age 15 and a median age of onset of 10 years [2]. PRS progresses slowly over a period of 2-10 years. Congenital cases occur but are uncommon. The younger the patient, the more severe the course of the symptoms [5]. Epidemiological reports indicate an annual incidence of 0.3-2.5 cases per 100,000 population, with a higher incidence among females, resulting in a male-to-female ratio of 1:3 [2].

PRS frequently becomes complicated by affecting neuronal systems, resulting in neurological and/or ophthalmic symptoms [5]. The most common neurological complications were described as epilepsy, headaches, and trigeminal neuralgia [4]. The etiology and pathophysiology of PRS remain largely unknown. Reports indicate that 21% of PRS patients also have scleroderma [6], and 24-34% develop the condition following trauma [7]. Potential causes include autoimmune disorders, trauma, infections, and sympathetic nerve dysfunction, necessitating a multidisciplinary team approach.

Mandibular osteomyelitis is fundementally the infectious inflammation localized to the mandibular bone marrow. Clinical feature of mandibular osteomyelitis is repetitive occurrence of chronic inflammation observed frequently with fistula and/or sequestrum formation [8]. The predominant cause is bacterial invasion originating from dental or periodontal sources [9]. Trauma, systemic factors as well as irradiation and medication can also be causative [8].

While reports exist of traumatic injury triggering PRS, to the best of our knowledge, after the database has searched up to November 2025, there are no reports of PRS occurring concurrently with mandibular osteomyelitis. This is the first reported case of concurrent mandibular osteomyelitis and PRS.

## Case presentation

The patient is a 46-year-old woman who presented with purulent discharge from a skin fistula in the right mental region as her chief complaint. Her medical history included localized scleroderma, diffuse type, with no significant family history.

**Figure 1:**
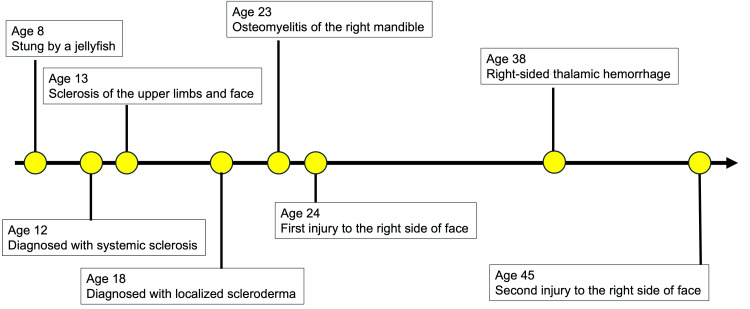
Clinical history of this case prior to visiting our hospital

According to the patient’s narrative history related to localized scleroderma, at the age of eight years, she was stung by a jellyfish somewhere on her body (Figure 1).

The wound scarred over and did not heal as expected. Two years later, at the age of 10, dermal sclerosis presented in her right forearm skin and spread to her fingers. At this point, the first premolars of both upper and lower jaws were extracted for orthodontic treatment. The orthodontic treatment proceeded without any issues.

At the age of 12, she visited a medical doctor to consult about the scarred wound and was diagnosed with patchy scleroderma. The symptoms continued to progress. By age 13, skin hardening and purplish-red changes had spread to the right fingers, upper limbs, and face (Figure 1).

At age 16, she was transported by ambulance after experiencing spastic-like pain and muscle weakness in her right thigh. Blood tests showed positive antinuclear antibodies at a 320-fold dilution; however, the skin biopsy revealed no abnormalities, and progesterone therapy was ineffective. Over the next two years, she was hospitalized three times. Treatment with nonsteroidal anti-inflammatory drugs (NSAIDs), antiepileptic drugs, and steroids proved ineffective. Two years later, at the age of 18, the skin on the right side of her face had hardened. Pain and cold sensations were also noted in the right mandible and temporal bone (Figure 1). She was diagnosed with localized scleroderma, diffuse type, by the rheumatology department.

At age 20, the patient presented to another hospital with right mandibular pain as the chief complaint and underwent extraction of the right lower third molar. The initial healing of the extraction socket was observed to be failing; however, it subsequently healed without any obvious issues thereafter.

At the age of 23, alveolar bone exposure was observed in her right lower jaw. A fistula was observed on the skin at the lower border of the right mandible (Figure 1). The patient was diagnosed with right mandibular osteomyelitis, and a debridement was performed to remove as much necrotic bone as possible. It was considered to be caused by periodontitis. No further bone exposure was observed after this treatment.

A year later, at age 24, she sustained an injury to the right side of her face, extending to the chin, due to a fall (Figure 1). Infected root canal treatment was performed on the right mandibular canine. Necrotic bone separation in that area, along with tooth mobility in the right lower lateral incisor and canine, was noted, gradually worsening. Temporary splinting of the right lower lateral incisor and the canine was performed, starting at age 27. The patient subsequently discontinued her outpatient visits. At age 29, she was taking 2.5mg of prednisolone (PSL) orally. Her right lower lateral incisor and canine were extracted.

At age 38, she suffered a right thalamic hemorrhage and was treated by a neurosurgeon. She underwent outpatient rehabilitation for left-sided paralysis (Figure 1). At the age of 42, the anti-ribonucleoprotein (RNP) antibodies, anti-centromere antibodies, and anti-topoisomerase I antibodies (Scl-70 antibodies) were all negative; however, oral PSL therapy continued.

**Figure 2:**
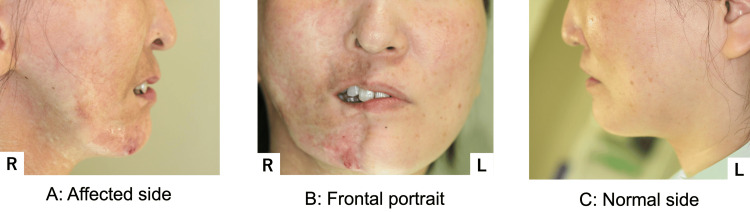
Facial appearance at the initial examination (A) Photograph taken from affected side; (B) Fontal photograph; (C) Photograph taken from normal side Facial appearance was asymmetrical, with atrophy observed from the right forehead to the right mental region.

At age 45, she fell again, injuring her mental region. She presented due to worsening suppuration and visited our hospital (Figure 1). At initial presentation, her facial appearance was asymmetrical, demonstrating atrophy from the right forehead to the right mental region (Figure 2).

**Figure 3:**
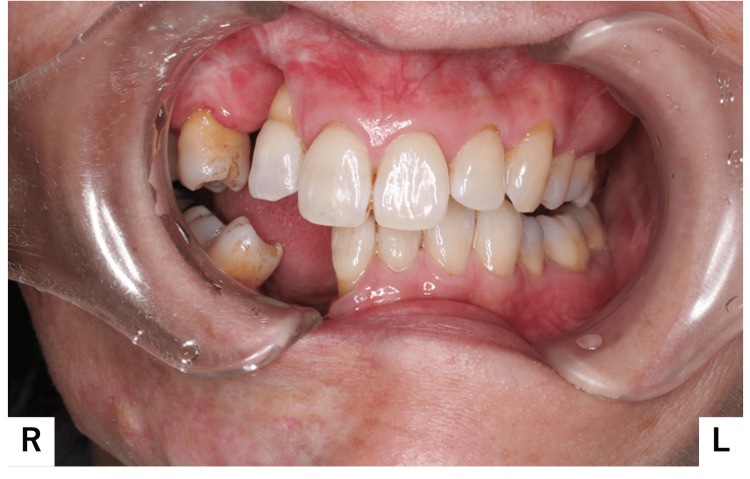
Intraoral photograph Occlusal contact in the right molar region was lost.

Closure of the right lip was difficult. A fistula and purulent discharge were noted on the right side of the chin; Horner's sign, indicative of upper sympathetic nerve damage, was absent. Intraorally, the buccal mucosa and gingival coloration were normal, and no dental abnormalities were noted; however, occlusal contact was lacking in the right molar region (Figure 3).

**Figure 4:**
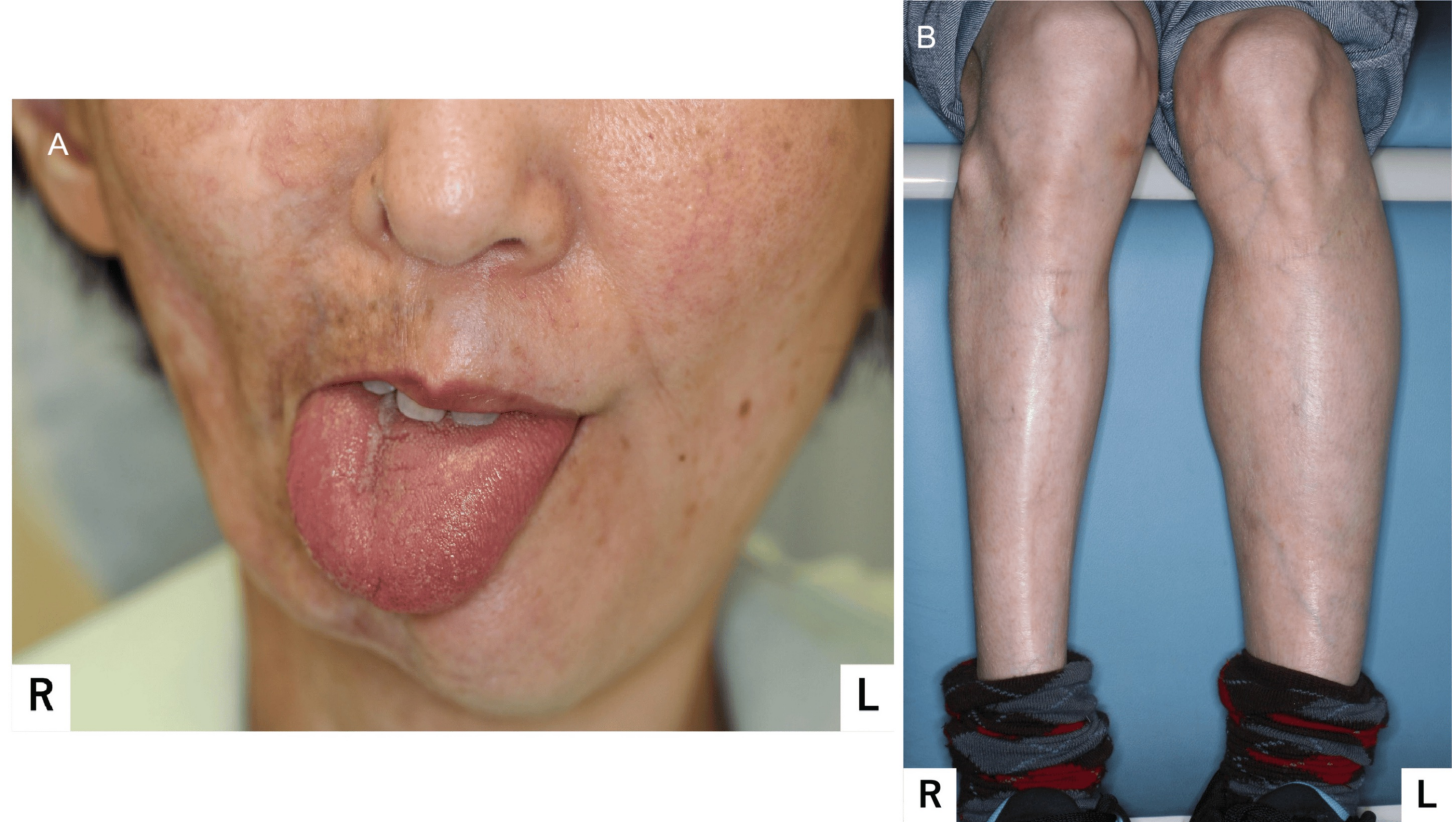
Tongue and general physical findings (A) Tongue on protrusion deviated to the affected side; (B) Atrophy of the right lower limb was observed.

Additionally, marked atrophy was observed in the right parotid papilla and sublingual tubercle. The tongue exhibited atrophy from the right margin to the tip, demarcated by the median sulcus. During protrusion, a deviation to the right was observed (Figure 4A). No sensory or taste abnormalities were noted, and the mucosal coloration appeared normal. Right-sided atrophy was observed, extending from the upper limbs and trunk to the lower limbs (Figure 4B).

**Figure 5:**
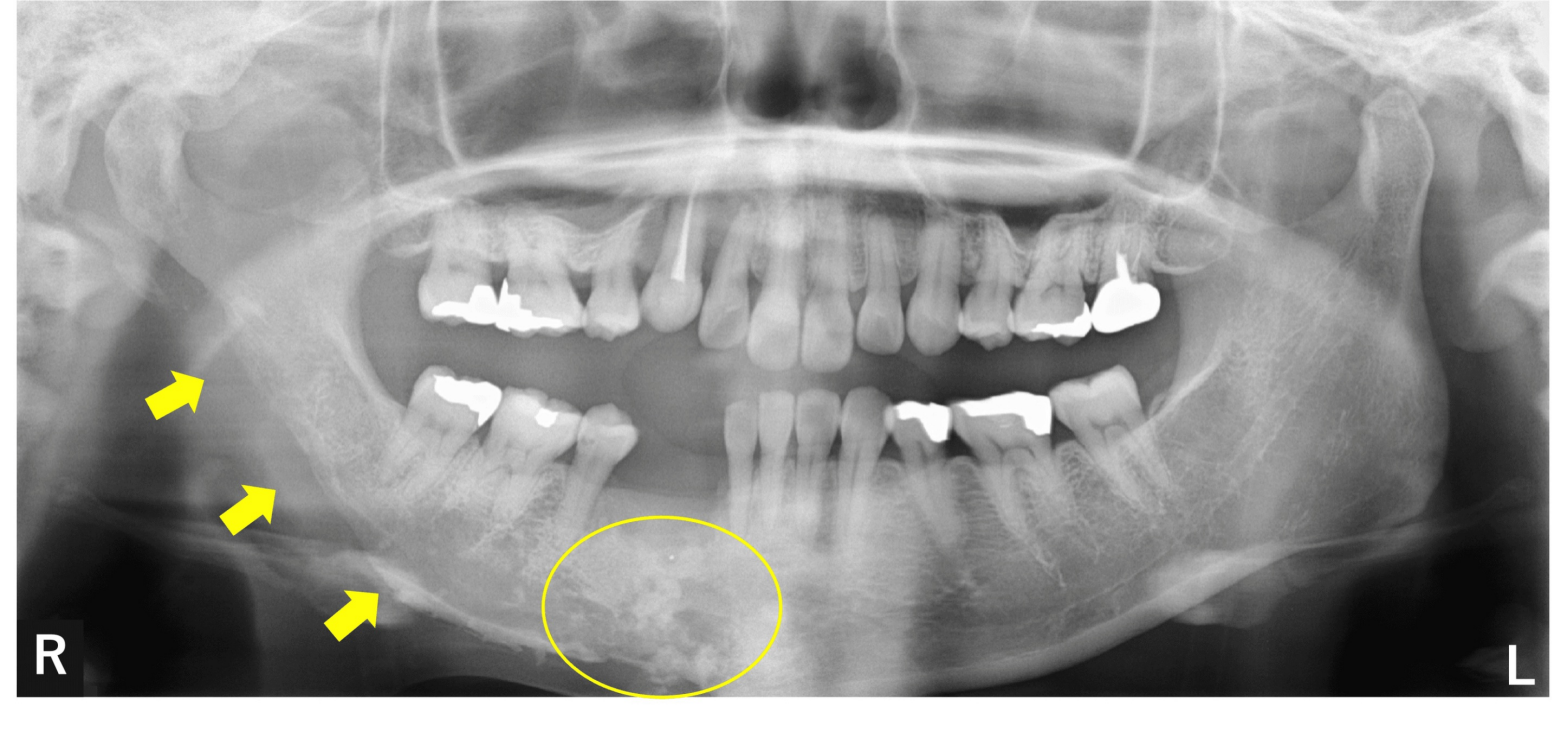
Panoramic X-ray showing radiolucency in the right mandibular bone areas (yellow arrows) and multiple punctate radiopaque areas in the mandibular bone (circle) area

Panoramic X-ray studies revealed significant radiolucency extending from the right mandibular ramus to the right mandibular angle and the mental region (Figure 5, yellow arrows). Multiple punctate radiopaque areas were noted in the regions corresponding to the missing right mandibular lateral incisor, right mandibular canine, and right mandibular first premolar (Figure 5, yellow circle).

**Figure 6:**
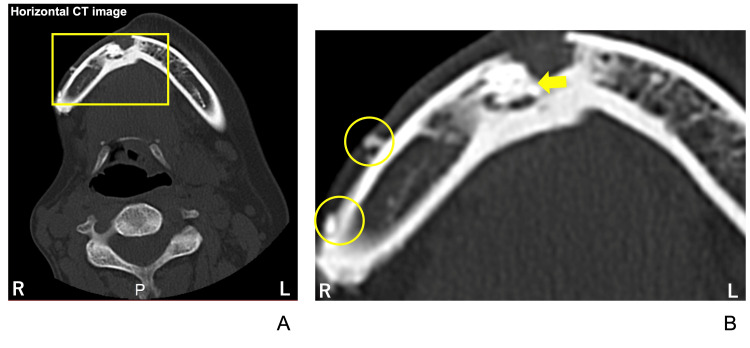
Horizontal CT images illustrating jawbone lesions The yellow square in A indicates the cortical bone defects observed in the right mandible, which are shown as a magnified view in B. Yellow circles and an arrow indicate several small bone fragments and a separated sequestrum in the body of the mandible, respectively.

Horizontal CT sections revealed atrophy of the right facial side. Partial discontinuity of the buccal cortical bone was noted, with sclerosis and necrotic bone findings observed from the mental region to the right mandible (Figure 6A). Additionally, several small bone fragments (Figure 6B, yellow circles) and a separated sequestrum (Figure 6B, yellow arrow) were identified on the buccal side of the right mandible. Bone fragments were also noted laterally to the right mandibular condyle at the temporomandibular joint level.

**Figure 7:**
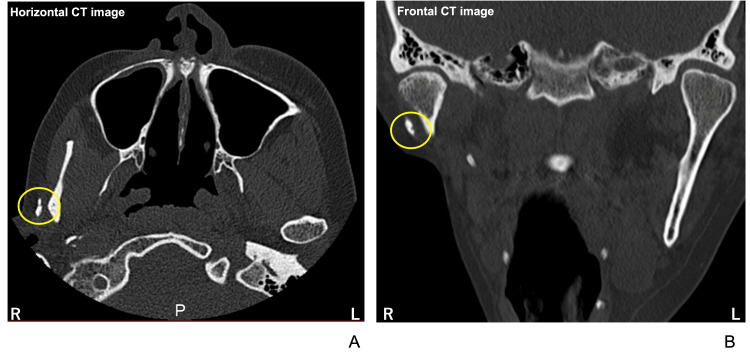
CT images at the maxillary sinus level (A) and the temporomandibular joint level (B). Yellow circles indicate the small bone fragments.

A cortical bone defect was identified in the lateral cortical bone of the right mandibular condyle on the frontal view (Figure 7).

**Figure 8:**
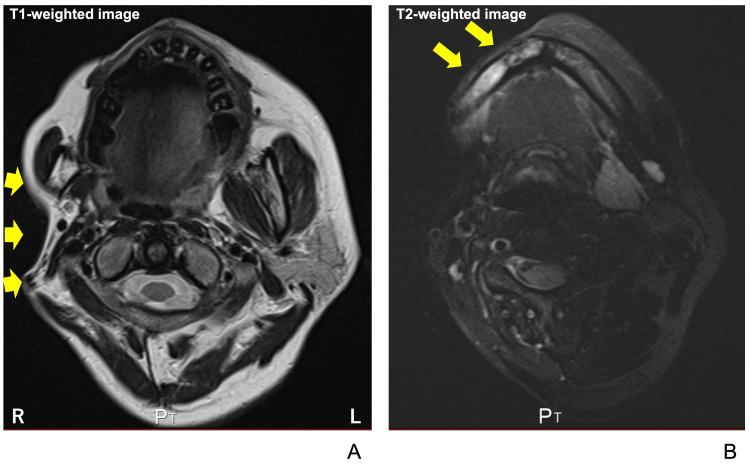
Horizontal MRI findings (A) T1-weighted MRI at the maxillary level; Yellow arrows indicate atrophic features of the parotid gland and masseter muscle, (B) T2-weighted MRI at the mandibular level, Yellow arrows indicate high signal intensity in the right mandible.

On T1-weighted MRI, marked atrophy of the right buccal fat pad, masseter muscle, and parotid gland was observed (Figure 8A, yellow arrows). Additionally, fat-suppressed T2-weighted images at the mandibular body level showed high signal intensity in the right mandibular body (Figure 8B, yellow arrows). 

Based on these findings, the diagnosis was progressive unilateral facial atrophy; PRS and chronic osteomyelitis of the right mandible.

**Figure 9:**
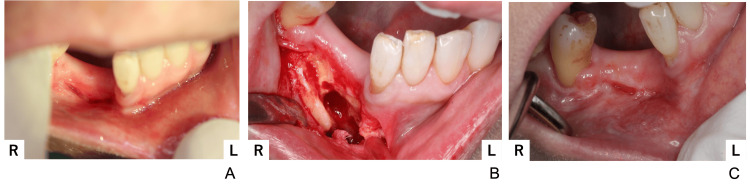
First surgical findings (A) Preoperative picture; a fistula and purulent discharge were observed, (B) Intraoperative picture; partial discontinuity of the buccal bone was observed after removing necrotic bone, (C) Postoperative picture; the wound was completely closed.

Debridement of necrotic bone was performed on the right mandible via the intraoral approach. A fistula and purulent discharge were noted at the buccal-gingival junction corresponding to the right mandibular lateral incisor and canine regions (Figure 9A). Antimicrobial susceptibility testing was performed on the purulent discharge. *Staphylococcus aureus* resistant to penicillin G (PCG), ampicillin (ABPC), erythromycin (EM), and clindamycin (CLDM) was detected. Necrotic bone was removed as completely as possible intraorally. Histopathological examination revealed granulation tissue predominantly composed of inflammatory cells such as neutrophils, lymphocytes, and plasma cells extensively infiltrating fibrous tissue, with partial inclusion of necrotic bone. The buccal bone lost its continuity in some areas (Figure 9B). The wound was closed entirely; however, an abscess was again noted at the same location 18 months later (Figure 9C).

Two years after the initial visit to our hospital, at the age of 47, a decision was made to perform extensive extraoral debridement of necrotic bone under general anesthesia. The blood test results at this time are shown in Table 1.

**Table 1:** Blood test before surgery Slight WBC and CRP increase were observed. RBC: red blood cell; WBC: white blood cell; Hb: hemoglobin; CRP: C-reactive protein; AST: asparate transferase; ALT; alanine aminotransferase, BUN; blood uria nitrogen, Cre; creatinine, eGFR; estimated glomerular filtration rate

Items	Results	Unit
RBC	4.37	×10^6^/µL
WBC	7.23	×10^3^/µL
Hb	12.0	g/dL
CRP	0.15	mg/dL
AST	20	U/L
ALT	19	U/L
BUN	13.4	mg/dL
Cre	0.4	mg/dL
eGFR	129.4	

**Figure 10:**
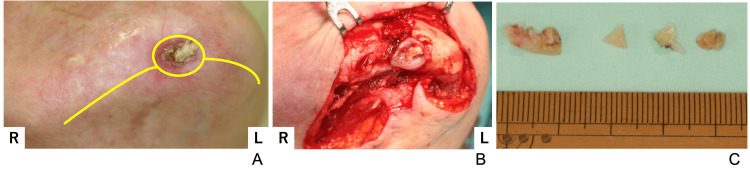
Second surgical findings (A) Preoperative picture; the incision line was placed below the mandibular border and around the periphery of the abscess, (B) Intraoperative picture; multiple free bone fragments were observed in the buccal cortical bone, (C) Photograph of the bone fragments; bone fragments measuring 5-10 mm were removed.

Before surgery, pus was collected and tested for antimicrobial susceptibility. At this time, the patient developed mandibular osteomyelitis and recurrent abscess. Due to the patient's circumstances, making frequent outpatient visits difficult, broad-spectrum antibiotics were administered until the scheduled surgery date. Oral fluoroquinolone antibiotics were initiated 20 days preoperatively. The antimicrobial susceptibility test results revealed that *S. aureus* was resistant to PCG, ABPC, EM, and CLDM, consistent with findings from two years prior. The abscess was incised around its periphery and beneath the mandibular border. Numerous small bone fragments were found around the buccal cortical bone of the mental region and were removed (Figure 10). 

**Figure 11:**
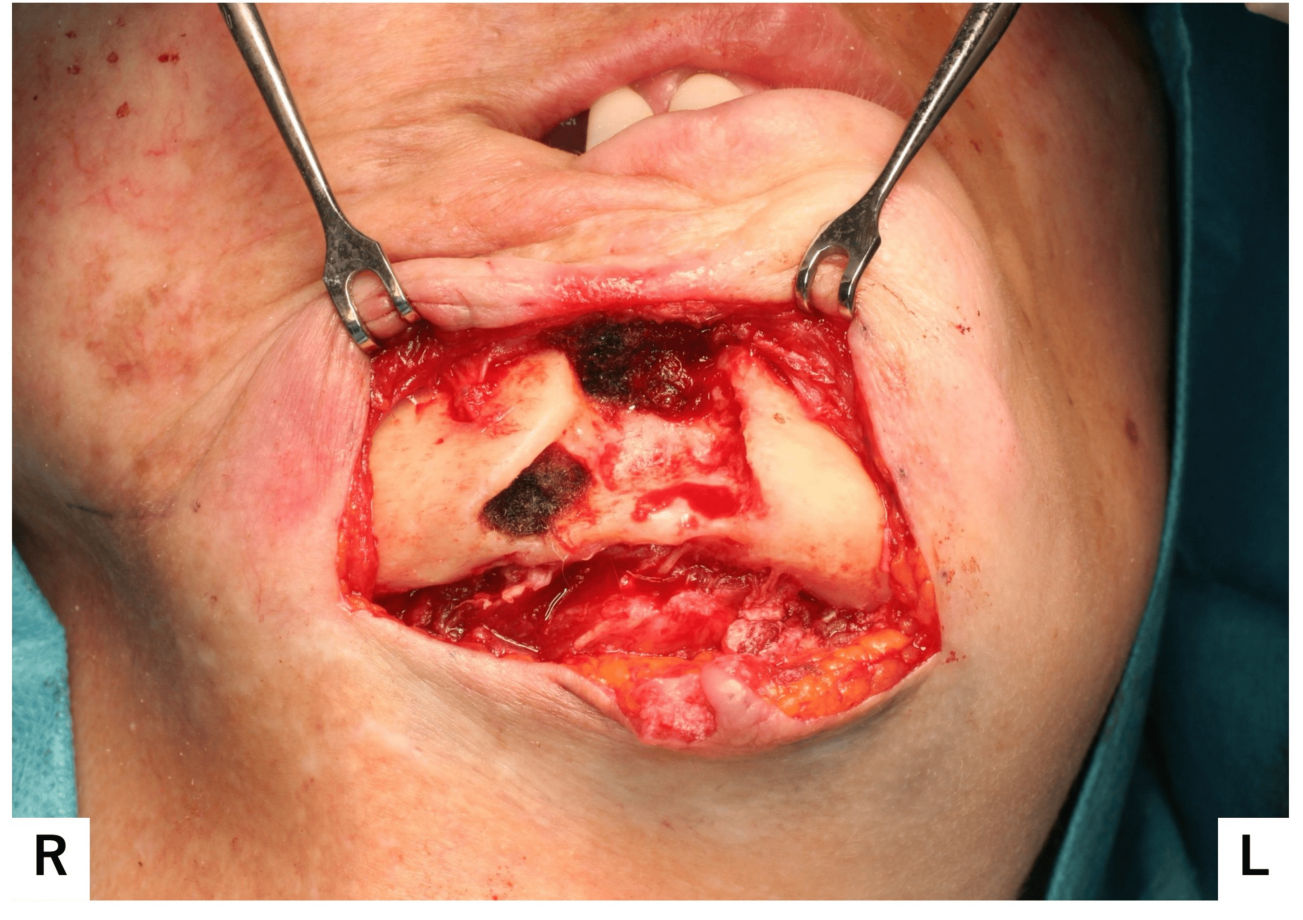
Post-extraction intraoperative image The buccal cortical bone of the mandible showed partial discontinuity; however, no fracture was observed.

Although the removal of necrotic bone and small bone fragments partially disrupted the bony continuity of the buccal cortical bone of the mentum, the procedure was completed without fracture (Figure 11).

Histopathological examination revealed necrotic bone containing inflammatory cell infiltration, such as neutrophils, in the superficial layer, along with surrounding non-necrotic bone tissue. Additionally, tissue, including skin, was submitted as a specimen, showing inflammatory granulation tissue with prominent inflammatory cell infiltration, including plasma cells, lymphocytes, and neutrophils, beneath the epidermis, and fragmentary necrotic bone was observed. Gram-positive cocci were observed by Gram staining. No actinomycete colonies were observed. Also, no filamentous organisms suggestive of actinomycetes or Nocardia, nor fungal components, were visible by Grocott staining. Thus, it was considered to be osteomyelitis of the mandible caused by *S. aureus*.

**Figure 12:**
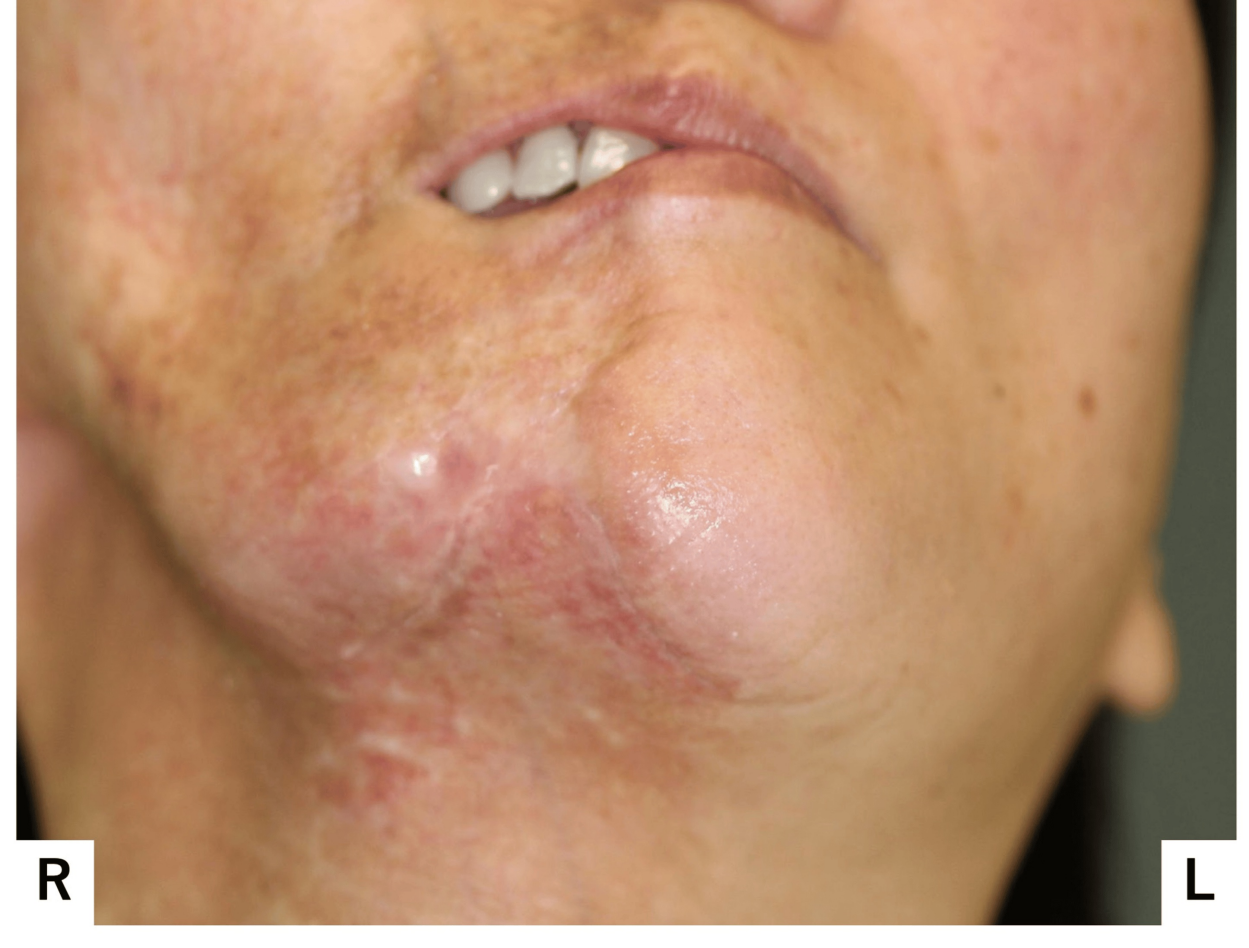
Postoperative finding There was no wound dehiscence, and the fistula disappeared.

Postoperatively, cefazolin was administered intravenously at a dose of 6 g/day for seven days because it was found to be susceptible in the antimicrobial susceptibility test. Sutures were removed from the surgical site on the 7th postoperative day. In accord with the patient's discharge on the eighth day after surgery, oral cephalexin antibiotics were continued until postoperative day 15. Oral antibiotic administration was discontinued as no postoperative infection was observed on postoperative day 15. On postoperative day 15, wound dehiscence was absent, and the fistula had disappeared (Figure 12).

At this time, the blood test also showed improvement in inflammation (Table 2).

**Table 2:** Blood test in 15 postoperative days WBC and CRP increase observed before operation was improved.

Items	Results	Units
RBC	4.49	×10^6^/µL
WBC	5.42	×10^3^/µL
Hb	12.5	g/dL
CRP	0.07	mg/dL
AST	25	U/L
ALT	25	U/L
BUN	8.2	mg/dL
Cre	0.36	mg/dL
eGFR	145.2	

The patient has undergone regular follow-up examinations for 10 years, and the surgical site has shown a favorable course without any problems. No facial atrophic progression or obvious recurrence of mandibular osteomyelitis has been observed. To ensure early detection and treatment of dental issues, regular follow-up examinations are conducted every few months.

## Discussion

Diagnosis of PRS primarily relies on clinical history, with correlations observed between imaging studies and histopathology. The differential diagnosis of PRS includes diseases characterized by facial hemiatrophy, such as localized scleroderma, hemifacial microsomia, Goldenhar syndrome, and cerebral hemiatrophy, including Rasmussen encephalitis and Sturge-Weber syndrome. In this case, the systemic and progressive nature led to the exclusion of all differential diagnoses other than localized scleroderma. PRS shares similarities with localized scleroderma but differs in that atrophy extends beyond the epidermis and dermis to subcutaneous tissues, including fat, muscle, bone, and cartilage (Table 3).

**Table 3:** Comparison of the characteristics of Parry-Romberg syndrome with that of Scleroderma diseases

Category	Parry-Romberg Syndrome	Localized Scleroderma (Linear/en coup de sabre)	Localized Scleroderma (Diffuse/Deep Morphea)
Main Symptoms	Progressive hemi-facial atrophy	Linear, band-like skin hardening (forehead/scalp/limbs)	Multiple or deep indurated plaques; possible muscle/fascia involvement
Skin Features	Sclerosis rare; mostly tissue loss	Well-demarcated sclerosis, pigment changes, alopecia	Marked skin thickening, may extend to deep tissues
Course	Gradual; often neurological	Usually local and limited	Extensive, may lead to joint/fascial symptoms
Lesion Distribution	Usually one side of the face (rare body/trunk)	Linear bands on face or limbs	Widespread, often symmetrical (trunk, limbs, deep tissues)
MRI/CT Findings	Facial/bone/cerebral hemiatrophy, white matter changes, calcification	Ipsilateral brain changes if craniofacial, local muscle/bone atrophy for limb lesions	Skin, fascia, muscle fibrosis and thickening; depth and activity well seen on MRI
Complications	Neurological signs (eg. epilepsy)	Contractures, visual disturbance (if periocular)	Joint limitation, functional impairment

Although a diagnosis of localized scleroderma was obtained in the rheumatology department, this case also demonstrated marked unilateral subcutaneous atrophy, leading to the diagnosis of PRS. CT and MRI findings of PRS in the face include varying degrees of hemiatrophy due to obliteration of the fat plane, ipsilateral deviation of the aerodigestive tract, and enophthalmos. We also confirmed marked atrophy of the right buccal fat pad, masseter muscle, and parotid gland were observed on T1-weighted MRI (Figure 8). Ipsilateral deviation of the aerodigestive tract can be observed in CT and MRI images (Figures 6-8). 

In some cases of PRS, dental hypoplasia has been reported to be associated, depending on the age of onset of the symptom [10]. When the onset occurs before the formation of the tooth crown and root, delayed eruption, tooth agenesis, root hypoplasia, and root resorption may occur. In this case, the onset occurred around the late teens, after the crown and root formation had been completed. Therefore, the intraoral findings and imaging studies did not reveal any morphological dental abnormalities. Furthermore, it is implicated not only in teeth but also in mandibular hypoplasia with spontaneous fractures [11]. Malocclusion develops as atrophy progresses with mandibular hypoplasia and oral hygiene deteriorating. Therefore, early orthodontic intervention and oral hygiene management are considered crucial. In this case, orthodontic treatment was performed, and the dentition was managed appropriately.

Treatment for PRS is primarily symptomatic, as no causal therapy exists due to the unknown pathogenesis. PRS is broadly classified into two types based on the depth of atrophy. Fat grafting is used when atrophy is confined to the dermis and subcutaneous tissue. When atrophy extends to cartilage or bone, a combination of fat grafting, bone grafting, microvascular free tissue grafting, or dermal grafting is employed [12]. In this case, we considered bone grafting using the fibula with the consultation of plastic surgeons. However, due to the extensive atrophy, it was determined that adequate skin coverage for wound closure could not be achieved. In addition to this, the patient declined high-risk reconstructive surgery. Therefore, the treatment of this case was limited to anti-inflammatory surgery for mandibular osteomyelitis.

The small bone fragments observed on CT and intraoperatively are thought to be mandibular bone-derived sequestra due to chronic inflammation from osteomyelitis. In this case, the prolonged periodontitis in the right mandibular lateral incisor and canine was involved in the development of mandibular osteomyelitis. Furthermore, since the patient has been diagnosed with scleroderma, she has been administered oral PSL for an extended period, which possibly led to osteonecrosis of the jaw. In addition, thin skin and connective tissue atrophy caused by PRS made the mandibular bone more prone to infection by trauma. Significantly, the osteomyelitis in this patient healed with pre- and post-operative antibiotic administration and appropriate local surgical therapy.

Several reports describe PRS cases triggered by trauma, infection, or inflammation. The patient in this case developed atrophic symptoms before experiencing facial trauma and local inflammation during late adolescence. Therefore, it cannot be excluded that being stung by jellyfish at the age of eight years might be a plausible contributing factor to the onset of the disease. Other proposed causes of PRS include autoimmune disorders, vascular dysregulation, and sympathetic nervous system dysregulation; however, supporting clinical research is lacking. This case demonstrated abnormal values of anti-nuclear antibodies only once during adolescence; however, these values returned to normal later on. Anti-nuclear antibodies are the most commonly associated serological finding in 25-52% of cases of PRS [2]. Another previous report suggested that autoantibodies seen in PRS are the result of pathogenic mechanisms that have been described in other forms of scleroderma [13]. Therefore, it is suggested that autoinflammatory changes are involved in the progression of PRS in this case. However, it is not clear whether the autoinflammatory changes are causative factors or readouts of the pathological progression.

A report indicates lymphocyte infiltration in the trigeminal neurovascular bundle, which is considered a cause of facial atrophy, along with abnormalities in the vascular endothelium and basement membrane [14]. This points to vascular dysfunction as a possible cause, though the mechanism behind lymphocyte infiltration remains unclear. An experimental animal model of unilateral cervical ganglion resection in rats demonstrated mild unilateral facial atrophy and a histological reduction of subcutaneous fat two months after the surgery [15]. Resection of the upper cervical sympathetic ganglion in rabbits, dogs, and cats also observed facial atrophy, including bone atrophy and ocular atrophy [16]. These animal model findings suggest that dysfunction of the sympathetic nerves disrupts peripheral blood flow. Prolonged neurovascular dysregulation can lead to poor tissue nutrition, resulting in atrophy. This case did not demonstrate clinical implications of superior cervical ganglion damage, as evidenced by the absence of Horner's signs. However, observing atrophic symptoms in almost half of the body of this patient, the involvement of hemi-dysfunction of peripheral neurovascular regulation cannot be excluded. This case also indicated the relevance of chronic inflammation concomitant with repeated trauma or covert autoimmune diseases in the progression of PRS.

The exact pathophysiology of PRS is still largely unknown. The occurrence and progression of the symptoms on only one side of the face or body robustly reminded us of a possible contribution of the peripheral nervous system (PNS). Recent understandings in neuroskeletal biology have elucidated the functional bidirectional pathways linking the PNS and bone and related tissues [17]. Neurocrine factors secreted peripherally from sensory and autonomic neurons have been known to regulate local tissue homeostasis, including the regulation of local stem cells, as well as neuro-vascular control of local nutrition [18]. Resection of sensory or autonomic neurons governing the facial area, such as the cervical ganglion or inferior alveolar nerve, in model animals appeared to at least partially mimic the symptoms of PRS [15,19]. These model animal studies support the idea of possible involvement of PNS in the pathophysiology of PRS. However, in this case, the symptom extended from the right side of the face to almost the entire right side of the body. Therefore, a higher neuronal regulation of the PNS can be considered involved in the pathogenesis. A recent finding in mice has elucidated that secretin-dependent signals in the ventromedial hypothalamus regulate bone and fat homeostasis through PNS [20]. If this finding can be extrapolated to humans, the right thalamic hemorrhage at age 38 in this patient is considered a critical event in the progression of the symptoms.

## Conclusions

We report a case of Parry-Romberg syndrome complicated by mandibular osteomyelitis. Due to its symptoms, PRS requires continuous follow-up and therapeutic intervention by a multidisciplinary team, including dental professionals. It needs the evaluation of the possibilities of local risks, such as dental problems leading to possible complications like mandibular osteomyelitis, and their consistent prevention. Although the precise cause of PRS remains unclear, animal studies suggest the involvement of dysregulation of the sympathetic nervous system and local blood flow. Given the limited number of animal studies reported thus far, future animal model research implemented with clinical findings is considered key to understanding the pathogenesis and developing causal therapy for PRS.
